# On Functional Module Detection in Metabolic Networks

**DOI:** 10.3390/metabo3030673

**Published:** 2013-08-12

**Authors:** Ina Koch, Jörg Ackermann

**Affiliations:** Molecular Bioinformatics group, Cluster of Excellence “Macromolecular Complexes”, Johann Wolfgang Goethe-University Frankfurt (Main), Institute of Computer Science, Robert-Mayer-Strasse 11-15, Frankfurt (Main) 60325, Germany; E-Mail: j.ackermann@bioinformatik.uni-frankfurt.de

**Keywords:** metabolic networks, functional module, Petri net, t-invariant, Fourier-Motzkin algorithm, elementary mode, maximal common transition set, t-cluster, minimal cut set, community, Q-modularity

## Abstract

Functional modules of metabolic networks are essential for understanding the metabolism of an organism as a whole. With the vast amount of experimental data and the construction of complex and large-scale, often genome-wide, models, the computer-aided identification of functional modules becomes more and more important. Since steady states play a key role in biology, many methods have been developed in that context, for example, elementary flux modes, extreme pathways, transition invariants and place invariants. Metabolic networks can be studied also from the point of view of graph theory, and algorithms for graph decomposition have been applied for the identification of functional modules. A prominent and currently intensively discussed field of methods in graph theory addresses the Q-modularity. In this paper, we recall known concepts of module detection based on the steady-state assumption, focusing on transition-invariants (elementary modes) and their computation as minimal solutions of systems of Diophantine equations. We present the Fourier-Motzkin algorithm in detail. Afterwards, we introduce the Q-modularity as an example for a useful non-steady-state method and its application to metabolic networks. To illustrate and discuss the concepts of invariants and Q-modularity, we apply a part of the central carbon metabolism in potato tubers (*Solanum tuberosum*) as running example. The intention of the paper is to give a compact presentation of known steady-state concepts from a graph-theoretical viewpoint in the context of network decomposition and reduction and to introduce the application of Q-modularity to metabolic Petri net models.

## 1. Introduction

The knowledge of biochemical networks, in particular of metabolic networks, increases daily with the capabilities of new upcoming high-throughput technologies to measure all the participating molecules and the relations between them. This enables us to construct large and complex models for many pathways of different species. In particular, modeling of metabolism helps us to understand biological function. 

A prerequisite for a quantitative model is the complete knowledge of metabolite concentrations and reaction constants and/or rates or, at least, a critical amount of them. However, in most cases, quantitative data in sufficient amounts and of high quality are rare and only available for rather small metabolic systems. This situation motivated the development of qualitative methods, which enable us to analyze statements on functional behavior and dynamic properties of the system without any knowledge of the kinetic parameters. 

Metabolism is commonly understood as a system of interacting and hierarchically organized functional *modules* [1]. Scale-freeness with the appearance of super-hubs, e.g., *ATP* or *NADH*, are typical features of metabolic networks [2]. The evolutionary reason and advantage of this organization structure is a topic of ongoing controversial discussions; see, for example, [2,3]. Currently, bioinformatics takes up the formidable challenges of characterizing the structural properties common in different metabolic systems and of identifying functional modules and their hierarchical organization. Many concepts, methods and algorithms emerge for network validation, decomposition and reduction. All are based on mathematical grounds and allow rigorous statements, even though the running time behavior becomes an issue for large networks. 

Graph-theoretical methods are based on topological properties, mainly connectivity, and do not account for stoichiometric relations or steady-state conditions. Such non-steady-state methods have been developed in various scientific fields, for example, in physics [4], social science [5], economy [6], marketing [7,8], production processes [9] and communication [10]. Many modularization techniques based on graph partitioning have been developed and studied over decades [11]. Recently, the Q-modularity introduced by Newman & Girvan [12] has boosted the research on community detection in graphs [13]. 

Most techniques have been developed for networks of one-to-one (*unipartite*) interrelations between components. These methods are suitable for biological interaction networks, such as protein-protein interaction in proteomics; see, for example, [14]. However, for reaction systems, such as metabolic pathways, it is beneficial to consider bipartite graphs, where metabolites cover the passive part, and the enzyme-catalyzed reactions, the active part of the system. This distinction enables a unique and exhaustive examination of the concurrent processes inherent in biological networks. The bipartiteness of graphs is a typical, intuitive feature in all complex networks [15], thus, also, in biochemical networks. Because Petri nets own bipartiteness by definition, Petri net theory is a suitable mathematical formalism for an appropriate description of metabolic networks [16]. Moreover, many mathematically proven methods exist for Petri nets, such as decomposition algorithms [17,18] or reduction techniques [19,20,21,22]. 

The literature in this field of ongoing research is extensive, and we abstain from giving a representative overview. The aim of this paper is, first, to present known steady-state methods for network decomposition from a graph-theoretical point of view; second, to introduce the application of Q-modularity to metabolic networks; and third to give a compact and understandable review on module detection discussed from both perspectives, with and without the steady-state assumption. 

In the paper, we aberrate from the traditional division into *Methods* and *Results* sections, because we partly present known concepts, but from a different point of view, in order to explain the new concepts. Thus, the organization of the paper is method driven. We start with the description of computer science terms of computability. Afterwards, we continue with a recapitulation of steady-state network decomposition methods and their application to metabolic systems, including a brief consideration of network representation as hypergraphs and bipartite graphs, the definition of Petri nets and a detailed explanation of the Fourier-Motzkin algorithm for invariant computation. Addressing graph-theoretical concepts, we define and discuss communities, Q-modularity and network reduction. In this context, we consider the use of functional modules for network verification and reduction. To illustrate the concepts for network decomposition and reduction, we apply a small biochemical running example. Finally, we summarize and give conclusions. 

## 2. Complexity Definitions of Algorithms and Problem Classification

In practice, we are interested in developing algorithms with the shortest possible running time. In computer science, problems formalized as algorithms are classified according to their running time behavior. This makes the formal estimation of running times of algorithms essential, including the development of a unique notation. We consider the running time dependent on the size of the input data and want to estimate the evolution of the computing time for big sizes of input data. Distinguishing the worst case, the best case and the average case, the worst case is of general interest and mainly applied. 

For pairwise sequence alignment, the size of the input data is defined by the sequence length; for multiple sequence alignment, the number of sequences to be compared needs to be included, as well. For graph-theoretical problems, the number of vertices, *n*, and edges, *m*, define the size of the input data. Now, we have to find a mathematical function that behaves similarly to the running time function, representing an upper, lower or tight bound. Commonly, the Landau notation [23] is used to denote asymptotic upper bounds (*O* and *o* notation), lower bounds (*Ω* and *ω* notation) and tight bounds (*Θ* and *θ* notation). As the *Big-O notation* for the worst case is most widely used, we explicitly give its definition. For a more detailed description we refer, for example, to [24]. 

**Definition 1 (Big-O notation [24]) :** *Let* *f(n) be the mathematical function that describes the behavior of our running time function. For a given function,* *g(n), we denote* *O(g(n)) as the set of functions with* *O(g(n)) =* *{* *f(n): there exists positive constants,* *c and* *n_0_, such that* *O* *≤* *f(n)* *≤* *cg(n) for all* *n* *≥* *n_0_* *}.*

The complexity theory classifies problems according to their running time behavior in the worst case. Algorithms, whose running time grows not faster than *O*(*n^a^m^b^*) with the exponents, *a* and *b*, as small as possible, are favorable. Problems, whose algorithms exhibit such a polynomial behavior, are classified to be in the complexity class, P (polynomial). Problems for which no polynomial-time algorithms are known, but whose solutions can be verified in polynomial time, belong to the complexity class, NP (non-deterministic polynomial). Problems like the Traveling Salesman, Boolean Satisfiability or Linear Programming are in NP. These problems are also called NP-complete. NP-complete problems are decision problems in NP and as hard as any other problem in NP. If there would exist a polynomial algorithm for one NP-complete problem, then every problem in NP would also have a polynomial-time algorithm. Then, the question, “P = NP?”, would have been solved and, thus, a fundamental problem in computer science. For a list of NP-complete problems in graph theory, we refer to [25]. NP-hard problems are at least as hard as any NP-complete problem, but do not have to be in NP. There exists many other subclass definitions for special problems. One of these definitions that we will need is the class, EXPSPACE, which is solvable with *O*(2*^p^*^(^*^n^*^)^) memory, where *p*(*n*) is a polynomial function of *n*. 

In practical applications, the complexity class of a task gives a reasonable indicator for the chance of success when we search for solutions in large graphs. Please keep in mind that the complexity class describes the worst-case scaling property. The simplex algorithm for linear programming represents a well-known example. It has an impressive record of running fast in practice, despite having exponential-time complexity when applied to a hard problem [26,27]. Note that the complexity class for the averaged scaling behavior is an independent (and interesting) question of its own. We will touch on the issue of complexity and computability later, again. 

The rather long and growing list of NP-complete problems motivated the development of alternative concepts, such as DNA computing [28,29], quantum computing [30] and membrane computing [31]. However, a discussion of the capabilities and limitations of these concepts are outside the scope of this work. 

## 3. Network Diagrams: Hypergraphs and Bipartite Graphs

**Figure 1:**
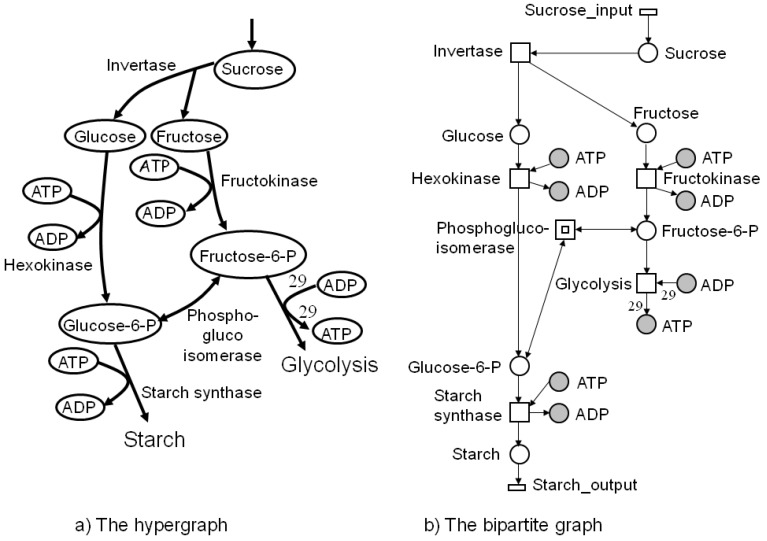
Part of the central carbon metabolism in young potato tubers (*Solanum tuberosum*) in the hypergraph representation on the left side and, on the right side, the corresponding bipartite graph as a Petri net. The metabolites are modeled as places and the reactions as transitions, which are labeled by the corresponding enzyme names. Transitions without pre-places or post-places model the interface of the system to its environment and are drawn as flat rectangles. Edges only exist between vertices of different types. Additionally, we see two other vertex types, which were introduced for a clearly arranged layout. The filled places stand for *logical* or *fusion* vertices. Logical places of the same name represent exactly one vertex in the underlying graph structure. A transition depicted by two nested rectangles stands for a *hierarchical* transition, meaning that it covers a subnetwork; here, the forward and backward reaction of the transition, *phosphoglucoisomerase*. If the edge label is not explicitly indicated, the edge weights are equal one. The transition, *glycolysis*, is enabled if there are at least one molecule or mole of *fructose*-6-*P* and 29 molecules or moles of *ADP* and produces 29 molecules or moles of *ATP* when it fires.

Graph-theoretical representations are widely applied to illustrate networks. For biochemical networks, these graphs are usually directed. Traditionally, biologists and physicians use the *hypergraph* representation; see Figure 1a. A hypergraph consists of a finite set of vertices, representing metabolites, and a finite set of *hyperedges*, denoting an arbitrary number of reactions that transform metabolites. In metabolic networks, a hyperedge covers one reaction, which is usually named after the enzyme that catalyzes this reaction. Figure 1a illustrates a hypergraph representation of a part of the central carbon metabolism in young *Solanum tuberosum* (potato tubers). The edges are weighted by an integer number that corresponds to the stoichiometric coefficient of the chemical reaction. For example, the hyperedge, *glycolysis*, in Figure 1a represents the underlying stoichiometric equation: 

*Fructose*-6-*P* + 29 *ADP* → 29 *ATP*.

**Figure 2:**
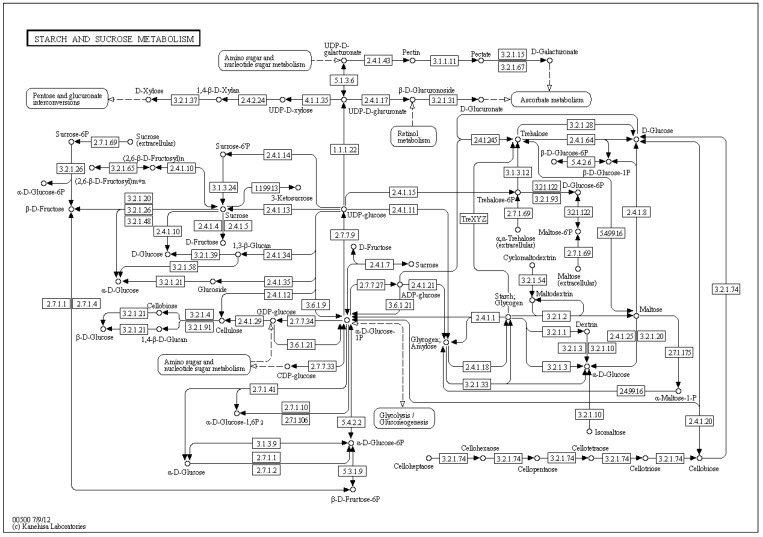
The KEGG [32] reference map, number 00500, of the starch/sucrose metabolism depicts a bipartite graph. The circles correspond to the metabolites, and the edges represent the enzyme-catalyzed reactions, where the enzyme is denoted by its EC-number in rectangles. The edges are directed and carry no information on stoichiometry.

The delineation of a metabolic reaction system as a *bipartite* graph is more detailed. Bipartite graphs are widely used in computer science. In bipartite graphs, two types of vertices exist, whereby edges are only allowed between vertices of different type, *i.e*., the edges separate the vertex set into two vertex sets. Researchers in biology and medicine are accustomed to metabolic pathway maps of the KEGG database [32] (see Figure 2) and, hence, inclined to apply bipartite graphs for visual representation. 

## 4. Petri Nets

*Petri nets* (PN) have been defined by Carl Adam Petri to describe systems with causal, concurrent processes [33]. PN are directed, bipartite graphs. The concept is developed under the strong division into passive and active system elements represented by two vertex types, the set of places, *P*, and the set of transitions, *T*. The vertices are connected by directed edges, defining a flux relation, *F*: ((*P* × *T*) ∪ (*T* × *P*)) → ℕ_0_. An edge never connects vertices of the same type, *i.e*., edges divide the set of vertices into two disjunct vertex sets. For an example, see Figure 1b. The metabolites are modeled as places and the reactions as transitions, which usually carry the name of the catalyzing enzyme. Transitions without pre-places or post-places model the interface of the system to its environment and are drawn as flat rectangles. Additionally, we see two other vertex types, which were introduced for a clearly arranged layout. The filled places stand for *logical* or *fusion* vertices. Logical places of the same name represent exactly one vertex in the underlying graph structure. Two nested rectangles stand for a *hierarchical* transition, hiding subnetworks. In Figure 1b, the nested rectangle covers the forward and backward reaction of the transition, *phosphoglucoisomerase*. If the edge label is not explicitly given, the edge weight equals one. Places can carry movable objects, the *tokens*. The distribution of tokens over all places defines a certain *system state*. The flow of tokens describes the dynamics of a system. The *marking*, *m* : *P* →ℕ_0_, determines the number of entities (e.g., molecules or moles) of each metabolite (place) and describes the current state of the metabolic network. 

Because tokens can be interpreted in different ways, for example, as objects of manufacturing or financial processes or as the number of moles or molecules, the token flow can be interpreted in various ways, strongly dependent on the application field. In metabolic networks, we consider a flow of substances, whereas in signal transduction networks, we consider a flow of signals, *i.e*., information. A token flow may take place if a transition is *enabled* or *activated* and *operates* or *fires* according to a specific *firing rule*, producing a new system state. In Figure 1b, the transition, *glycolysis*, is enabled if there are at least 29 tokens of *ADP* and one token of *fructose*-6-*P*, and the capacity of the corresponding post-place is large enough to accept the produced 29 tokens of *ATP*, additionally to the existing marking. In most cases, places with unbounded, *i.e*., infinite, capacity are defined. 

**Figure 3:**
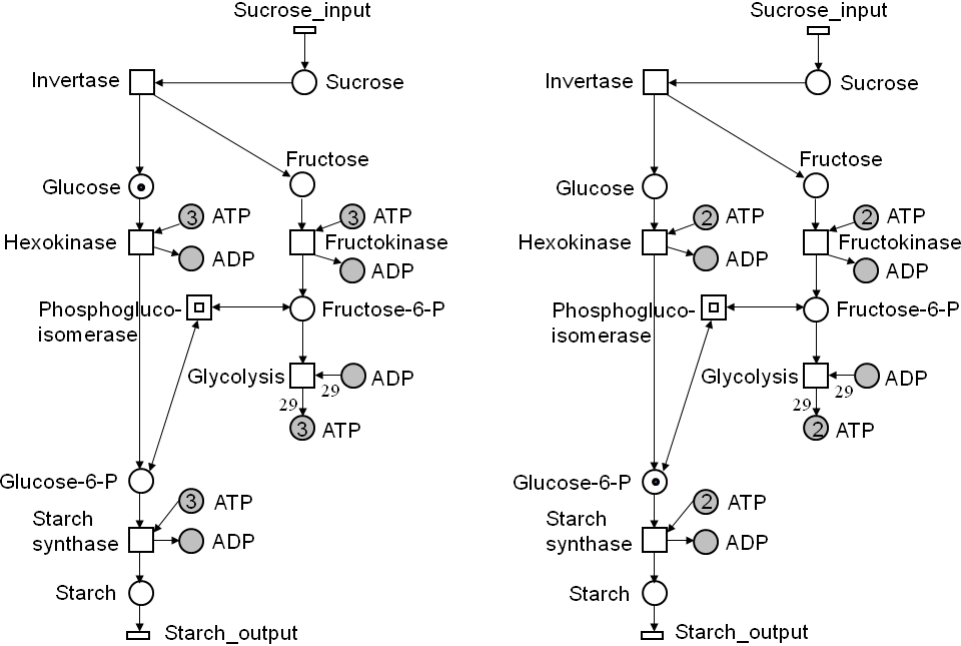
Two possible states of the Petri nets (PN) of Figure 1. On the left side, the place, *glucose*, carries one token, and the place, *ATP*, depicted by three *logical* places, carries three tokens. Thus, the transition, *hexokinase*, is enabled or has concession and can fire. After firing (right side), one token of glucose-6 phosphate (place *glucose*-6-*P*) was produced, consuming one token of *ATP* (place *ATP*). One token of sucrose was generated by firing of the transition, *sucrose input*, which is always enabled.

In this paper, we consider the untimed firing rule of classical place/transition nets (P/T-nets). That means that firing, *i.e*., token movement, takes no time. The number of consumed and produced tokens is defined by the weights of the corresponding edges to the pre-and post-places, respectively, of the firing transition. Note that the total number of consumed tokens must not be equal to the total number of produced tokens. Thus, a PN may not conserve the total number of tokens in the system. Figure 3 shows two states of the PN in Figure 1. On the left side, place *glucose* carries one token and the place, *ATP*, depicted by three *logical* places, three tokens. Thus, transition *hexokinase* is enabled and can fire. After firing (on the right side), one token of glucose-6 phosphate is generated, consuming one token of *ATP*. Moreover, one token of sucrose has entered the system by the firing of transition, *sucrose input*, which is always enabled. To explore the entire dynamic behavior, all reachable states have to be computed. 

### 4.1. Reachability Analysis

**Figure 4:**
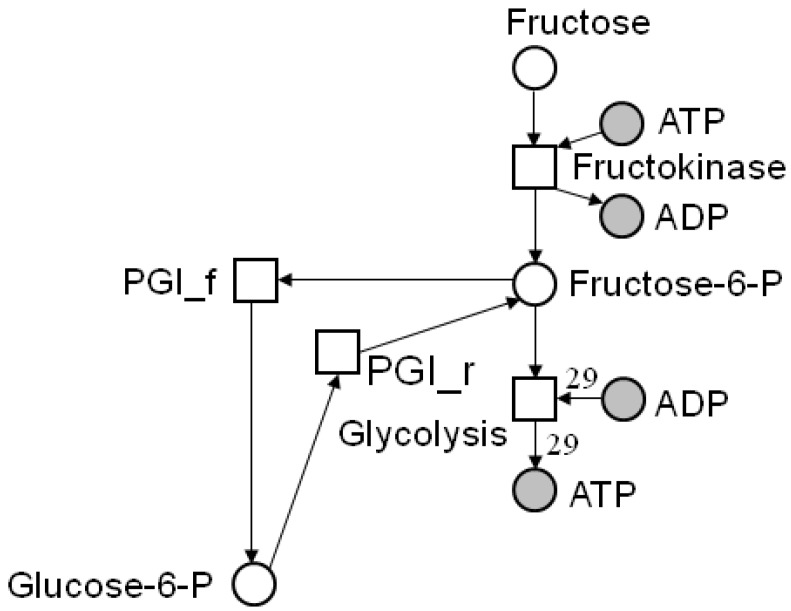
Two conflicting transitions in a part of the network of Figure 1. The place, *fructose*-6-*P*, has two post-transitions, *PGI_f_* and *glycolysis*, which compete for the tokens on the place, *fructose*-6-*P*. For the reachability analysis, we have to consider the two cases: (1) transition *PGI_f_* fires first or (2) transition *glycolysis* fires first.

The reachability analysis aims to enumerate and investigate all possible system states starting from an arbitrary initial marking. In the analysis, we have to follow all alternatives of firing in the case of conflicts and concurrency. This results in a *semi-ordered* (*partial-ordered, interleaving*) semantics that reflects the nondeterministic choice of the processes to be executed. In the case of simulation, we have to decide, for example, which transition of two or more conflicting transitions fires in which order. Figure 4 illustrates a small subnet of the central carbon metabolism in young potato tubers of Figure 1. The place, *fructose*-6-*P*, has two post-transitions, *PGI_f_* and *glycolysis*, which both compete for the tokens on the place, *fructose*-6-*P* . For the reachability analysis, we have to consider the two cases: (1) transition *PGI_f_* fires first or (2) transition *glycolysis* fires first. To represent all possible states and the transitions that cause the respective new states, we define the *reachability graph* RG. The vertices of an RG encode system states, each defined by a certain token distribution on all places. The directed edges, labeled by the reaction whose firing induces the change of the system state, indicate the direction of the state transformations. 

Usually, a standard graph-theoretical algorithm, called *Breadth-First Search* (BFS) (see, for example, [24]), is used as basis for the computation of the RG. This algorithm explores all vertices of a graph, starting with an arbitrary vertex and all its neighbors. The visited vertices are labeled, such that they are not processed again. The algorithm continues with the unvisited neighbors, until all vertices of the graph have been explored. Thus, for example, all connected components of a graph can be determined. The BFS algorithm runs in linear time in *O*(*m* + *n*), where *m* and *n* are the number of vertices and edges, respectively. Here, the BFS examines all enabled transitions as neighbors of the considered state. The exponentially growing number of system states can lead to a *state space explosion*.Here, the BFS examines all enabled transitions as neighbors of the considered state. In biology, even for small networks with up to 20 places and 30 transitions, the state space may become very huge. Therefore, in the last few years, special data structures, e.g., binary decision diagrams (BDD), have been developed to cope with the state space explosion [34]. 

### 4.2. Incidence Matrix and Stoichiometric Matrix

Let us consider a sequence of reactions, *s* =(*t_i_*_1_,*t_i_*_2_,...,*t_in_*), also called *firing sequence*, which changes the marking of the system, such that:

*m_new_* = *m_old_* +Δ*m* (1)

and

Δ*m*= *Cτ* (2)

The number of occurrences of reactions, *t_k_* ∈ *T*, in the firing sequence, *s*, is given by the component, *τ_k_* =#*t_k_*, of the frequency vector, *τ*: *T*→ ℕ_0_. We call *τ* a *Parikh vector* of the sequence, *s*. Originally, Parikh vectors have been defined for context-free languages, indicating the number of occurrences of a letter in a word [35]. 

Generally, an *incidence matrix*, *C*, describes the relationships between two sets of objects, for example, *T* and *P*, which corresponds to the columns and rows of the matrix, respectively. The matrix entry, *C*(*x*, *y*), is nonzero, if *x* and *y* are related, and zero, otherwise. For a weighted, directed, bipartite graph with the edge weights, *w_tp_* and *w_pt_*, the two sets are defined by the two vertex types, *i.e*., *t* ∈ *T* and *p* ∈ *P*. The two possible directions, forward and backward, of an edge are specified by the numbers, *d_f_* =1 and *d_b_* = −1, respectively. An entry, [*x*, *y*], in the incidence matrix is given by *d_f_w_pt_* and determines the change of the token number in a place, *p*, after the firing of a transition, *t*; see Table 1. In such a way, we describe the effect of a sequence of firing transitions (reactions) on the marking of the system by the incidence matrix, *C* : *P* ⊗ *T* → ℤ. Table 1 illustrates the incidence matrix, *C*, of the PN in Figure 1 covering eight places and nine transitions. The token change of metabolites in the marking on the places is then given by:

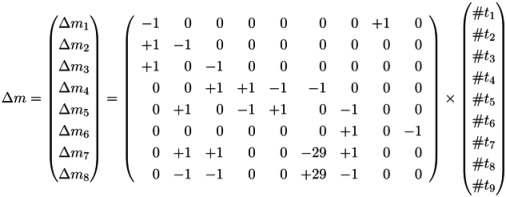
(3)


**Table 1:** The incidence (stoichiometric) matrix for the network in Figure 1. *p_i_* stands for a metabolite (place) and *t_j_* for a reaction (transition).

*C*	*t*_1_: Inver-tase	*t*_2_: Hexo-kinase	*t*_3_: Fructo-kinase	*t*_4_: Phospho-glucosio-merase_f	*t*_5_: Phospho-glucosio-merase_b	*t*_6_: Glyco-lysis	*t*_7_: Starch synthase	*t*_8_: Sucrose input	*t*_9_: Starch output
*p*_1_: Sucrose	−1	0	0	0	0	0	0	+1	0
*p*_2_: Glucose	+1	−1	0	0	0	0	0	0	0
*p*_3_: Fructose	+1	0	−1	0	0	0	0	0	0
*p*_4_: F6P	0	0	+1	+1	−1	−1	0	0	0
*p*_5_: G6P	0	+1	0	−1	+1	0	−1	0	0
*p*_6_: Starch	0	0	0	0	0	0	+1	0	−1
*p*_7_: ADP	0	+1	+1	0	0	−29	+1	0	0
*p*_8_: ATP	0	−1	−1	0	0	+29	−1	0	0

The firing of transition *t*_8_ (*sucrose input*) produces a new token of sucrose on *p*_1_; see Figure 1. In this case, the Parikh vector, *t*, has solely one nonzero component, #*t*_8_ =1, and we yield:

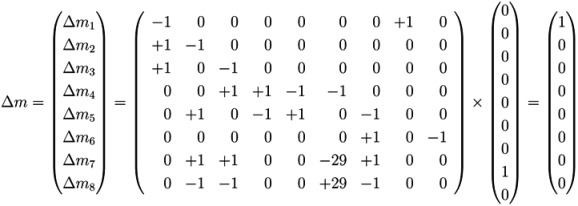
(4)
We may use the incidence matrix, *C*, to compute the changes of token (metabolite) numbers, resulting from the firing sequence of transitions (reactions) (*t*_4_,*t*_5_).

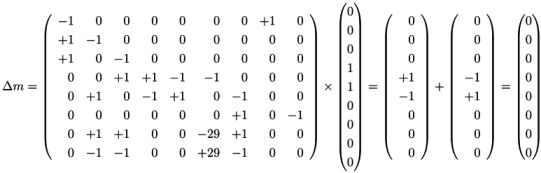
(5)
The change of metabolites produced by this firing, sums up to zero. In terms of PN theory, the two reactions, *t*_4_ and *t*_5_, form a *transition-invariant*. One of these two reactions, let us say, *t*_4_, may fire spontaneously and drive the systems away from the steady state, but the other reaction, *t*_5_, of the transition invariant can compensate for the effect of firing of *t*_4_. Such stochastic fluctuation is a natural and inherent property of a metabolic reaction system, in which all reactions are constantly active and the time-dependent state of the system fluctuates around an ideal steady state. 

### 4.3. Invariants

Let us now consider the invariant properties of the system. The invariants hold in every system state reachable from an arbitrary, initial marking. We define invariant properties for the active and the passive part of the system. Considering the active part and the equation system:

*C t* = 0 (6)

we define a nontrivial, nonnegative integer solution, *t*, and name the solution vector, *transition-invariant* or *t-invariant*. The solution, *t*, has to be an integer, because we consider discrete objects—the tokens, and nonnegative—because any sequence of firing transitions (reactions), *s* =(*t_i_*_1_,*t_i_*_2_,...,*t_in_*), gives rise to an integer and nonnegative Parikh vector. Parikh vectors with negative components are senseless in the biological context. 

Let *C^T^* be the transposed incidence matrix. Considering the passive part, we define the nontrivial, nonnegative integer solution, *p*, of the equation system:

*C^T^p* = 0 (7)

and call it *place-invariant* or *p-invariant*. 

The solution space of such linear equation systems is, in general, unbounded, *i.e*., infinite. However, we are interested in a finite solution set, from which we can compute all possible solutions by positive integer linear combinations of the solution vectors. Such a set is given by all *minimal* solutions of the invariant equations, where *minimal* means: for an invariant, *x*, there exists no invariant, *z*, whose support is part of the support of *x*:

∄ invariant *z* : *supp* (*z*) ⊆ *supp* (*x*) (8)

and the largest common divisor of all entries of *x* is one. The *support* of *x*, written as *supp*(*x*), contains the set of the nonzero entries of a vector, *x*. In the following, we consider minimal, nontrivial, nonnegative t-and p-invariants, shortened as t-invariants or TI and p-invariants or PI, respectively. 

Invariant properties have important applications in systems biology. P-invariants represent a set of places whose weighted sum remains always constant, thus representing a conservation of substances. T-invariants describe a cyclic firing behavior, because the firing of all transitions of a t-invariant leads back to the initial marking, forming a cycle in the RG. The TI represents basic pathways in biochemical networks at steady state and describes, thus, the basic network behavior. Before explaining the application of invariants in more detail, we first want to discuss their computation. 

### 4.4. Fourier-Motzkin Elimination Method

The Fourier-Motzkin elimination method (FM) [36,37] is a classical algorithm for solving equation systems with minimal, nontrivial, nonnegative integer solutions, *i.e*., the computation of t-invariants. The working principle of the FM can easily be demonstrated for the network in Figure 1. Initially, we construct a table that consists of the transposed incidence matrix and the |*T*|×|*T*| identity matrix: 

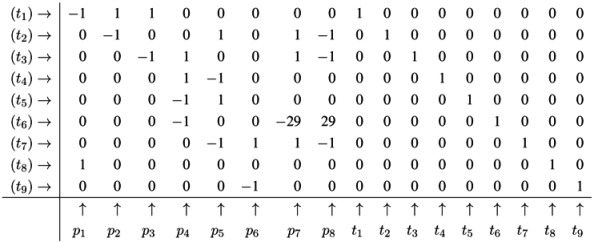

We find one column for each reaction (*t*_1_,*t*_2_,...,*t*_9_) and one column for the change of metabolites in each place (*p*_1_,*p*_2_,...,*p*_8_). We can read the table as follows: Each line describes a sequence of reactions and its effect on the metabolites. The first line corresponds to the sequence (*t*_1_), because the entry for reaction, *t*_1_, is one, and the entries for all other reactions are zero. This sequence of reactions (*t*_1_) removes one metabolite of *p*_1_ and adds one metabolite of *p*_2_ and one metabolite of *p*_3_, because in the first line, the entry is −1 for *p*_1_, and the entries for *p*_2_ and *p*_3_ are both +1. The interpretation of the second to the eighth line is analog. The basic idea of the algorithm is to combine lines in such a way that the entries for all metabolites become zero. In the first step, we have to select a metabolite, let us say *p*_1_, and to construct the combinatorial diversity of all sequences of reactions that yield Δ*m*_1_ =0. Checking the column for *p*_1_, we find an entry, −1, in the first line (*t*_1_) and an entry, +1, in the eighth line (*t*_8_). Each of the reactions, *t*_1_ and *t*_8_, influence the metabolite, *p*_1_, and the only possible combination of these reactions that yields Δ*m*_1_ =0 is reaction *t*_1_ plus reaction *t*_8_. Consequently, we add the first line to the eighth line to get one new line with an entry zero for *p*_1_. We append this new line (*t*_1_,*t*_8_) to the table and delete the lines utilized to construct that new line. We get the new table: 

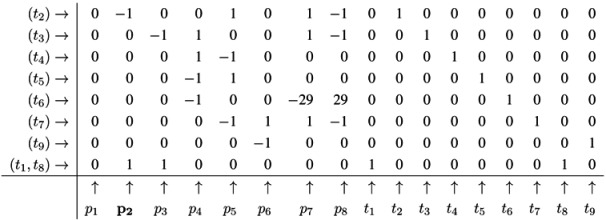

Note that all entries in the column for *p*_1_ are zero now. The new line (*t*_1_,*t*_8_) in the table has an entry, one, in the column for *t*_1_ and an entry, one, in the column for *t*_8_. Hence, the new line corresponds to the reaction sequence (*t*_1_,*t*_8_). According to the FM, we still have to check whether the new sequence of reactions is *minimal*, because the support of a new sequence may be a superset of the support of another sequence. In this case, the new sequence would not correspond to a minimal solution and would have to be eliminated from the table. In the particular case of reaction (*t*_1_,*t*_8_), the candidate solution is minimal. The FM algorithm proceeds with another metabolite that has nonzero entries in its column. For large networks, the metabolites should be chosen according to an advantageous heuristic [37]. For simplicity, we leave such heuristic aside and just choose the metabolite, *p*_2_, in the table above. In the column for *p*_2_, we find the entry, −1, in the line, (*t*_2_), and the entry, one, in the line, (*t*_1_,*t*_8_). Again, we can construct only one new combination that gives *p*_2_ =0. We construct the new line, (*t*_1_,*t*_2_,*t*_8_), by adding the first line, (*t*_2_), to the eighth line, (*t*_1_,*t*_8_). Then, we delete the utilized lines and append the new line: 

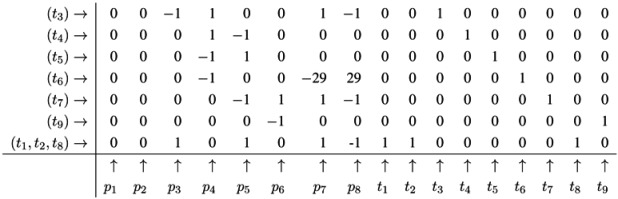

The new line corresponds to a minimal candidate solution, and the FM proceeds with another metabolite that still has nonzero entries in its column. Proceeding to the next step of the FM for metabolite *p*_3_, two lines are utilized to append a new (minimal) line, (*t*_1_,*t*_2_,*t*_3_,*t*_8_): 

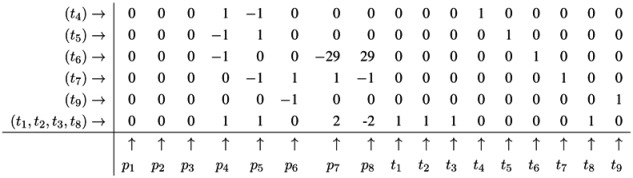

In the next step of the FM, the combination of the fourth line, (*t*_7_), with the fifth line, (*t*_9_), zeroes all entries for the metabolite on *p*_6_, and we yield: 

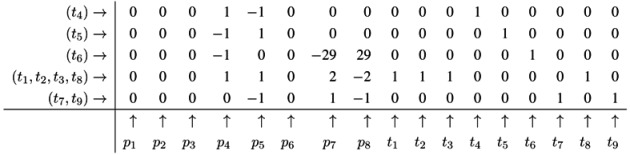

Note that, up to now, each step of the FM has reduced the number of lines in the table. Such a reduction of the number of lines is favorable, because, in general, the number of lines grows in each step of the FM. The growth can be exceptionally fast and presents a serious problem, known as the state explosion problem for the computation of all minimal solutions of Diophantine equation systems. We choose (carefully) a network as an example, for which the FM does not run into the state explosion problem. The next step of the FM for metabolite *p*_4_ shows that the number of lines does not have to decrease for each step. Proceeding with metabolite *p*_4_, we find two positive and two negative entries in the column for *p*_4_. The algorithm has to construct the combinatorial diversity of all solutions, which zeroes the entries in the column for *p*_4_. Such combinations are the first line, (*t*_4_), with the second line, (*t*_5_), the first line, (*t*_4_), with the third line,(*t*_6_), the forth line, (*t*_1_,*t*_2_,*t*_3_,*t*_8_), with the second line, (*t*_5_), and the forth line, (*t*_1_,*t*_2_,*t*_3_,*t*_8_), with the third line, (*t*_6_). In this way, we construct four new (minimal) lines and get the new table: 

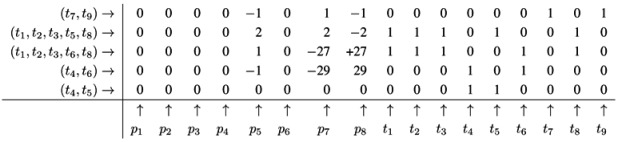

The next step for metabolite *p*_5_ results in the table: 

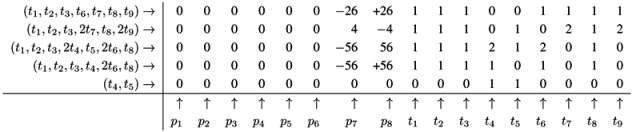

Applying a further step for metabolite *p*_7_ gives the final table: 

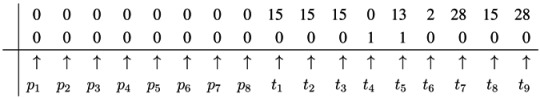

The entries for the metabolites, *p*_1_ to *p*_8_, have all to be zero in the final table. The entries for the transitions describe two t-invariants of the network. The t-invariant, {*t*_4_,*t*_5_}, represents a simple reversible reaction, and is called a *trivial* t-invariant. The more complex t-invariant, {15 *t*_1_, 15 *t*_2_, 13 *t*_3_, 2 *t*_5_, 28 *t*_6_, 15 *t*_7_,*t*_8_, 28 *t*_9_}, describes the basic functional pathway of the network in Figure 1b. It is easy to verify that all reactions are members of at least one TI, and hence, the network is covered by t-invariants CTI. 

Reactions that *cannot* be compensated by other reactions have to be discussed carefully for their biological relevance. Such reactions are strong indicators for missing reactions or errors in the model. The identification of a reaction that contradicts a steady-state behavior is a computational challenge for large metabolic models. Standard approaches are based on the computation of a minimal generator set of all TI. In general, the computation of all TI requires exhausting resources in terms of computer time and memory [38]. Several groups have developed advanced algorithms to speed up the computation of all TI, for example, the canonical basis approach by Schuster & Hilgetag [39], the nullspace approach by Wagner [40], the concept of bit pattern trees by Terzer & Stelling [41], and a parallel divide-and-conquer approach by Jevremovic *et al.* [42]. Even with all these methods and modern (super-)computers, only models of moderate size have been tractable until now. The number of TI of a metabolic network of moderate size can easily reach tens of millions [42]. This leads us to the next problem: how to interpret this huge amount of basic pathways. Which pathways are the most important ones? To give an answer, let us now consider first the CTI question without the computation of all t-invariants. 

### 4.5. The CTI Property

We want to define another property, which is helpful, in particular, to verify biochemical systems. This property represents a completeness condition which may be applied in network verification. If each transition belongs to at least one t-invariant, we say that the PN is *covered by t-invariants* (CTI). Accordingly, we call a PN to be *covered by p-invariants* (CPI), if each place is a member of at least one p-invariant. The CPI property can be used to decide boundedness, *i.e*., the finite number of tokens for all places. Only for bounded PN, a finite reachability graph can be generated. Though the CPI property is important for many questions, we will not consider it in more detail in this paper. 

#### 4.5.1. The CTI Question

Despite the fact that the knowledge of an even huge number of t-invariants is valuable and represents a prerequisite for more advanced analytical techniques, we want to decide whether a network is CTI without computing all TI. Since the set of all t-invariants describes a minimal set of all functional modes of the system at steady state, each transition should belong to at least one t-invariant. To show the CTI property for a PN, we have to find *one* integer solution, *t*, of the equation:

*C t*=0 (9)

with nonzero components (*t*_i_ ≥ 1,*i*=1, 2,..., |*T*|) or to exclude the existence of such a solution. Thus, to decide the CTI question without computing all TI, a less expensive strategy would be beneficial. 

Lipton [43] gives the proof that the reachability problem for vector addition systems requires exponential space in the worst case. Accordingly, the CTI decision problem is EXPSPACE-hard. For a vector addition system, (*s*, *e*, {*v*_1_,*v*_2_,...,*v_n_*}), of dimension *k*, the reachability problem reads: do vectors *w*_1_,...,*w_m_* ∈ℕ*^k^* exist, such that:

*w*_1_ = *s* (10)

*w_m_* = *e*, *and* (11)

*for each i*, *w_i_*_+1_ = *w_i_* + *v_j_*, *for some*+ *j*? (12)

We can easily see the equivalence to the CTI decision problem. Let the dimension, *k*, be the number of places, (*k* = |*P*|), theincremental change vectors, (*v*_1_,*v*_2_,...,*v_n_*) ∈ ℤ^ k^, the column vectors of the incidence matrix, *C*, the end vector, *e* ∈ ℕ*^k^*, sufficiently large and the starting vector, *s* ∈ ℕ*^k^*, the sum of the end vector, *e* ∈ ℕ*^k^*. Then, the change of metabolites, resulting from firing each transition once, is:

*s*= *e*+ *Cy* (13)

where in the Parikh vector, all components equal one (*y_i_* =1,*i*=1, 2,... |*T*|). Any solution of this vector addition problem represents a solution of Equation (9) and shows the CTI property of the network. 

### 4.6. Geometric Point of View

The CTI question and the concept of TI are closely related to the theory of convex cones. In this context, Schuster *et al.* [39] defined the *elementary flux modes* or *elementary modes* (EM), which correspond to the TI [44]. It is obvious that the set, 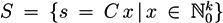, of all solutions of Equation (6) is a pointed convex cone. A network is CTI if and only if the effect of firing of all reactions can be compensated. Firing of all reactions in our example network in Figure 1 results in the following changes of metabolites:

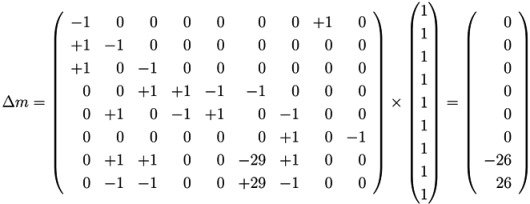
(14)
These changes of metabolites have to be compensated by a reaction of the net. Therefore, we have to find an element, *s*, in the convex cone, *S*, that compensates for Δ*m*, *i.e.*, *s* = −Δ*m*. Geometrically, the network is CTI if the vector:

*b*= −*C y* (15)

is inside the convex cone, *S*, and the Parikh vector, *y*, has all components equal to one (*y_i_* =1, *i* =1, 2,... |*T*|). We have to check whether *b* is inside (*b* ∈ *S*) or outside (*b* ∉ *S*) of the cone. Here, the Lemma of Farkas [45] provides a useful statement: The vector, *b*, is either inside of the convex cone, *S*, or it is possible to find a hyperplane, *S*_⊥_, that separates *b* from the convex cone. Such a separating hyperplane must be a tangent hyperplane to the convex cone, *S*. Without loss of generality, we can choose a surface normal, *s*_⊥_, that points into the same direction as the cone, *i.e*., the angle between *s*_⊥_ and all vectors in the cone is not greater than 90°:

*C^T^s*_⊥_ ≥ 0 (16)

This inequality can be expressed as:

*C^T^s*_⊥_ − *I_ν_* =0 (17)

where *I* is the identity matrix and *ν* is an arbitrary vector with nonnegative integer components. The surface normal, *s*_⊥_, determines a tangent hyperplane, *S*_⊥_ = {*s* ∈ ℤ*^k^*|*s^T^* *s*_⊥_ =0}. 

Now, we have to prove whether the vector, *b*, is located on the “wrong” side of the hyperplane, *i.e*., opposite of the convex cone, *S*. It turns out that the vector, *b*, is located opposite of the convex cone if a solution, (*s*,*ν*), of Equation (17) with nonzero positive components of *ν* ≥ 0 exists [46]. The nonzero components of *ν* identify the reactions not covered by TI. Applying this strategy to the network in Figure 1, we have to construct all solutions (*s*_⊥_,*ν*) of the dual system (17):

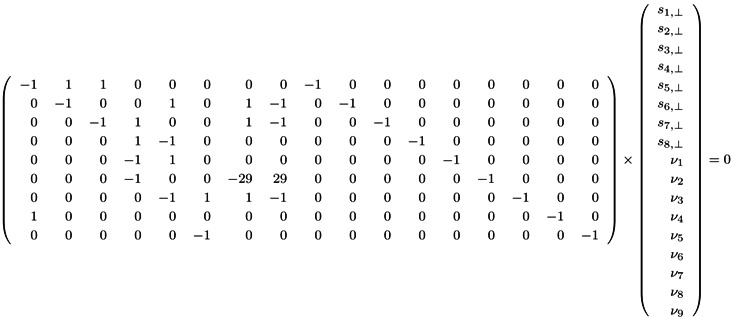
(18)
The FM can be again applied to construct all positive integer solutions, (*s*_⊥_,*ν*), of this system of the Diophantine Equation (17). In general, solutions of Equation (17) with zero components, *ν* =0, are called place-invariants in PN formalism [22] and describe the conservation law of metabolites [47]; see Section 4.3. Note that the computation of solutions of the system of the Diophantine Equation (6) and the computation of solutions of the dual system of the Diophantine Equation (17) are both EXPSPACE-hard problems [46]. 

## 5. Network Decomposition into Functional Modules

Functional modules are important for representing, understanding, reducing and verifying general networks. This is true, in particular, for biochemical networks, which are big and complex and for which an experimental validation can be difficult or is even not possible. Several definitions of functional modules have been proposed in various scientific fields. Definitions inspired by biology are mainly manually derived induced by biological knowledge. They often rely on the experience of the individual researcher. With the growing amount of data, the automatic detection of modules becomes of great interest. All known definitions are at least implicitly based on graph-theoretical properties. 

For biochemical systems, we distinguish between module definitions that are based on the steady-state assumption and definitions that ignore it. Both types of definitions are advantageous to solve specific biological questions. 

### 5.1. Steady-State Modules

The reactions (transitions) of each EM (TI) and the metabolites (places) in between, including the corresponding edges between them, build connected subnetworks that stand for a certain biological function. Thus, a subnetwork defined by a TI can be understood as a functional module. The careful evaluation of the biological interpretation of functional modules, often manually done, is part of proving the model for its correctness. There are many studies that provide exactly this kind of analysis. Some of them report the detection of new pathways that have been later experimentally validated. An example is the prediction of the glyoxylate pathway [48,49] and its validation [50]. Because the number of TI can grow exponentially, thousands to millions and more of TI can exist, even for middle-sized networks of two or three hundred vertices. To handle such a huge number of functional modules, further differentiation becomes necessary and was developed by several groups. We distinguish between methods that are based on the support of a TI vector and others that consider the actual numbers in the Parikh vector. 

#### 5.1.1. Support Vector-Based Methods

Methods based on the support vector do not explicitly take into account the integer numbers of the Parikh vector and, thus, implicitly ignore the stoichiometric relations. Instead, we consider the binary information of whether a reaction or enzyme (transition) is a member of a TI or not. An example of such a method to define modules are *minimal cut sets*. 

*Minimal Cut Sets (*MCS*)* [51]: MCS has been introduced to study the fragility of metabolic networks and possible knockout strategies to prevent or avoid a specific biological function. An MCS is defined as a minimal set of reactions (enzymes) that blocks, after its removal, all feasible, balanced fluxes that involve an objective reaction (enzyme). Applying the Lemma of Farkas, MCS can be computed without the computation of the TI [52]. 

The next two module definitions are suitable for large networks. Since our running example is too small to illustrate the usefulness of these definitions, we refer to examples in [53,54,55]. 

*Maximal Common Transition sets (*MCT*-sets)* [56,57]: Inspired by maximal common subgraphs, we summarize equal parts of the solution vectors into new sets, the MCT-sets. An MCT-set is defined by a set of reactions, {1,...,*m*}, in which each pair of reactions, *t_i_* and *t_j_*, with *i*,*j*∈ 1, ...,*m*, occurs in exclusively the same TI, such that:

*χ*_{__0__}_(*x_i_*) = *χ*_{__0__}_(*x_j_*) (19)

where *X* denotes the set of all TI *x*, with *i*, *j* ∈ 1, ...,*m*, and *χ*_{__0__}_ denotes the characteristic binary function, indicating if its argument equals zero. This grouping leads to maximal sets of transitions, where each set of transitions, *ϑ*, fulfills:

∀*x* ∈ *X*: *ϑ*⊆ *supp*(*x*) ∨ *ϑ*∩ *supp*(*x*) = φ (20)

Because of the exclusive membership of transitions, MCT-sets and the places and edges in between define disjunctive subnetworks. Thus, MCT-sets can be interpreted as building blocks, for example, in synthetic biology. Please note that the reactions of an MCT-set do not necessarily represent connected subnetworks, *i.e*., they do not necessarily form consecutive firing sequences. 

*T-clusters* [54,58]: Whereas MCT-sets define disjunctive subnetworks caused by the strong criterion of exclusiveness in their definition, we may wish to allow *overlapping subnetworks* with a broader, specific biological function. We define *t-clusters* based on hierarchical clustering methods, such as UPGMA or NEIGHBOR JOINING. As a distance measure, we use the Tanimoto coefficient [59]. The similarity between two t-invariants, *t_i_* and *t_j_*, is then:

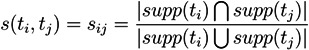
(21)
where *supp*(*t_i_*) and *supp*(*t_j_*) denote the support vectors of the t-invariants, *t_i_* and *t_j_*. The pair-wise similarity, *s_ij_*, expressed by this coefficient is transformed into a distance measure for dissimilarity, *d_ij_* [60]:

*d_ij_* =1 −*s_ij_* (22)

For a detailed description of clustering techniques, see, for example, [61].

The definition of the *best* number of clusters, which is a fundamental problem in unsupervised classification, is implemented as a user-defined parameter. Additionally, cluster validity measures can be applied to identify the number of clusters which “best” represents the intrinsic grouping of the data [62]. The *silhouette width* [63], which is computed as the average silhouette value over all data samples, seems to be a suitable measure for biochemical applications. The silhouette value, *S*, for an individual data sample, *i*, is defined as:

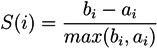
(23)
where *a*_i_ denotes the average distance between *i* and all the data samples in the same cluster and *b*_i_ denotes the average distance between *i* and all data samples in the nearest other cluster. In contrast to MCT-sets, subnetworks based on t-clusters can overlap. 

MCT-sets and t-clusters have been applied to metabolic systems, but also to signal transduction pathways [57] and gene regulatory networks [64]. An interesting biological interpretation is that the reactions of an MCT-set take place always together, *i.e*., the expression behavior of the participating genes should be similar. 

*ACoM (Aggregation around Common Motif)* [65]: Starting with a *common motif* defined as the set of transitions that belong to all TI as a seed, it will be extended according to specific rules. This seed motif is of determined length and is successively extended, until a certain threshold is reached. Similar to t-clusters, overlapping *aggregations of common motifs* were defined. 

*Elementary Flux Patterns* [66]: The concept of elementary flux patterns is similar to EM analysis. It explicitly takes into account possible steady-state fluxes through a genome-scale metabolic network when analyzing pathways in a subsystem. Thus, many EM can be computed in reasonable time, although not the complete set of all EM or TI. The concept of elementary flux patterns allows for the application of many EM-based tools to genome-scale metabolic networks. 

#### 5.1.2. A Parikh Vector-Based Method

*Enzyme subsets (ES)* [67]: Enzyme subsets are enzymes that always operate together in fixed flux distributions in all steady states of the system. In the context of Metabolic Control Analysis, groups of enzymes were introduced as *monofunctional units* or *super-enzymes* [68,69]. In monofunctional units, all Parikh entries of the TI, *i.e*., the ratios of (nonzero) frequencies of the reactions, have to be identical. This requirement represents a restrictive criterion for the definition of functional modules. 

### 5.2. Communities As Non-Steady-State Modules

Communities play a prominent role in a broad range of scientific fields, including, e.g., social science, economics, computer science, engineering, politics, and biology. Examples of communities are friends in a school class, readers of books sharing similar interests, electronic components to be placed together on a layout of a solid-state circuit board, co-authors of scientific articles, interacting proteins or words with similar associations. For an excellent review, we refer to the work of Fortunato [13]. Communities are intuitively understood as a group of members of a network. The members should have many connections within the community and only a few connections to vertices outside the community. Interrelations inside the communities should be dense and between the communities, sparse. The well accepted quality criterion, called *Q-modularity*, for a partition into communities is defined by:

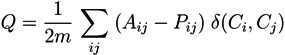
(24)
for a unipartite network with *m* unweighted undirected edges and communities, *C* [12]. The formula sums the entries of the adjacency matrix, *A*_ij_, over all pairs of vertices, *i*,*j*, in the same community. The Kronecker delta, *δ*(*C_i_*,*C_j_*), becomes one, if both vertices, *i* and*j*, are in the same community and zero, otherwise. The summation over *A_ij_* gives the number of edges inside of all communities and a number that cannot exceed the total number of edges times two. The pre-factor, 1/(2*m*), guarantees a value which is equal to or less than one. Each entry, *A_ij_*, is reduced by a probability, *P_ij_*, to find the edge, (*i*,*j*), by chance in an appropriately chosen statistical null model. A random network with identical distribution of the vertex degree leads to a simple sum over *n_c_* modules:

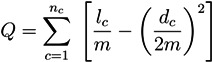
(25)
where *l_c_* is the number of edges in the module, *c*, and *d_c_* is the total sum of the vertex degrees in the module, *c*. This formula for the Q-modularity is not directly applicable to metabolic networks because metabolic networks are bipartite graphs with directed edges and edge weights. An easy way to apply the formula above would be to transform a metabolic network into a unipartite network with undirected and unweighted edges. This can be done in different ways, but a loss of crucial information (e.g., the direction of flow of metabolites) cannot be avoided. 

#### 5.2.1. Q-Modularity

**Figure 5:**
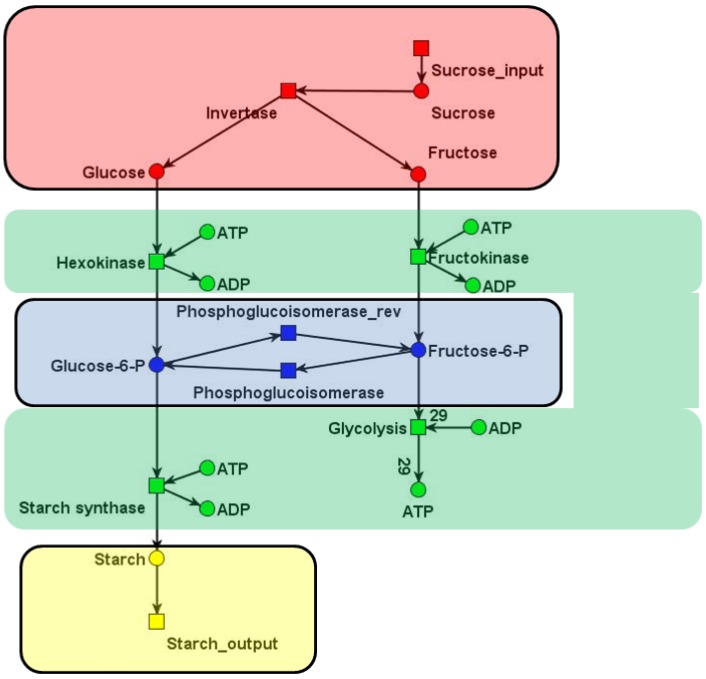
The structure of modules optimized using a genetic algorithm. Each colored subnet represents a community: the red subnet describes the sucrose uptake and cleavage by invertase; the green subnet covers all reactions, where *ADP* and *ATP* are participating; the blue subnet describes the only reversible reaction in the system, and the yellow one stands for the starch production.

A partition of a PN is given by disjoint modules, *C_i_*, with *i* =1,2,...,*n_c_*. The vertices of a module can be transitions and/or places. An appropriate formula for *Q*-modularity of metabolic networks has to consider the direction of edges within modules and between modules in a bipartite metabolic network [70]. Note that, to find modules for which the value of *Q* reaches its maximum is an NP-hard problem [13]. We apply a genetic algorithm to obtain an optimized structure of modules for metabolic networks. The value of the *Q*-modularity increases from generation to generation and reaches a maximum after a sufficient number of steps. Figure 5 shows an application of this algorithm to the network in Figure 1b. 

## 6. Application to Network Reduction and Verification

We have already discussed the complexity class of various methods for analyzing qualitative properties of metabolic networks. The search for the best possible partition in modules is an NP-hard task, and the CTI question is EXPSPACE-hard. For example, the rather medium-sized metabolic network of *Saccharomyces cerevisiae* with 63 metabolites and 117 reactions considered in Jevremovic *et al.* [42] has about 50 million TI. Keeping in mind this huge number of invariants and the extensive computational effort required to compute them, it seems to be hopeless to apply an invariant analysis to metabolic networks of thousands of reactions as published in current databases [71]. The computational effort may *explode* with the increasing number of network components. This *explosion problem* is a well-known drawback in practical computations. However, it is instructive to see how the explosion problem can be circumvented for networks using special networks properties: metabolic networks are usually expected to be scale-free; reaction chains appear often; there are super-hubs of metabolites playing an essential role for most reactions (e.g., *ATP*); many reactions are reversible and most likely have a small number of one or two input metabolites. Such properties make metabolic networks special and well-distinguishable from random networks or technical networks. 

**Figure 6:**
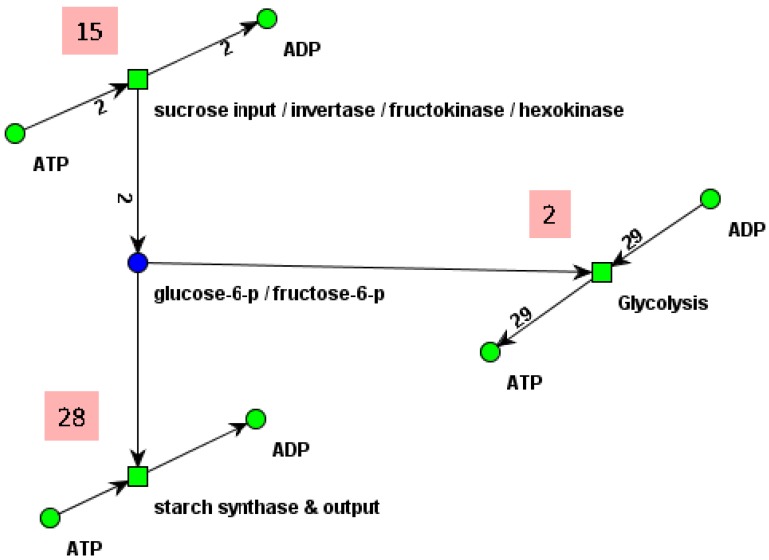
Reduced network of a part of the central carbon metabolism in young potato tubers (*Solanum tuberosum*) of Figure 1b. The original network, consisting of eight metabolites and nine reactions, is reduced by four common transition pairs (CTP) and one invariant transition pair (ITP). The three reactions of the reduced network represent the concerted action of several reactions of the original system. The places, *glucose*-6-P and *fructose*-6-P , lump together the metabolites, glucose-6 phosphate and fructose-6 phosphate. The reduced network is CTI and has only one TI. The frequencies of the individual reactions in the invariant are given by the numbers in the red colored rectangles. Note that the two minimal invariants of the original network (compare Section 4.4) can be constructed by an appropriate extension procedure [46]. The picture was generated using MONALISA [73].

It may, for example, be possible to reduce the computational effort to answer the CTI question by transforming a network into a smaller one. Thus, *network reduction* enables insights into coarse-grained structural properties of the network [19,20,21,22,72]. Useful network reduction techniques for the CTI question are transformations of networks that preserve the CTI property. These CTI-*conservative* reduction techniques are favorable to decide the CTI question for large networks. For most biological networks, a significant reduction of the computational complexity is possible. A typical kind of a reduction step is inspired by MCT-sets [57] or *enzyme subsets* [67] (see Section 5.1). The basic idea is that chains of reaction can be summarized to one reaction if they consist of *common transition pairs* (CTP). A CTP is a local structure of a place that has exactly one pre-transition and one post-transition. Intuitively, the pre-transition produces tokens on a place that can be removed by the post-transition only. Another local structure useful in this context is the *invariant transition pair* (ITP). An ITP is a reversible reaction, consisting of a forward and backward reaction. Figure 6 depicts an example for network reduction. For a detailed definition and discussion, we refer to [46]. 

The starting point for the analysis of a new constructed model should always be the theoretical verification of the model. Standard approaches are based on the condition that the model should have the ability to establish an equilibrium with the environment, i.e., external resources have to be supplied by the environment, and accumulating metabolites have to be discharged. We may find dynamic properties of a model that contradict such a steady-state behavior of the system. An iterative process of verification and remodeling is necessary to improve the model and to correct fundamental errors. Thus, laborious computations based on the mass action kinetics or stochastic simulation of a not validated and, possibly, erroneous model can be avoided. 

Metabolic networks are commonly described in terms of mass action kinetics, using kinetic parameters such as concentrations of the metabolites, reaction constants and rates. The steady-state behavior of the model may, in principle, be evaluated by applying bifurcation theory, local stability analysis and the theory of dynamical systems [74,75]. However, the nonlinear character and the high number of resulting equations hinder such an approach for most metabolic reaction systems, besides the fact that the kinetic parameters in most cases are unknown. Moreover, such a point of view of metabolism is well-satisfied only for well-mixed systems of large spatial dimension. Biological systems, for example, a cell or mitochondrion, are characterized by a complex spatial organization in a small volume. The assumption of well-mixed concentrations of freely diffusing proteins, complexes and small metabolites that react by mass action kinetics inside of a large macroscopic volume is obviously not always met for such systems. Even for small metabolites, the functional role of gradients of concentrations and non-diffusive transport processes (e.g., see the electron transport chain in mitochondria) hamper the application of mass action kinetics. The number of enzymes and metabolites are discrete, countable and not even nearly on the order of the Avogadro constant, *N_A_* =6.02214 × 10^23^. A theoretical description in terms of probability functions and solutions of the stochastic master equation would be more realistic to specify the fluctuation of species in the system [76,77]. Even at the steady state, the numbers of molecules are not constant, but fluctuate around average values, where the average number of molecules of a species depends on its chemical concentrations. 

## 7. Summary and Conclusions

The work aims to give an overview about important methods in both connectivity-based, as well as steady-state-based methods. In this paper, we report two types of approaches for functional module detection: those that are based on the steady-state assumption and those that are based on graph-theoretical methods without a steady-state consideration. The first one considers a bipartite graph representation of metabolic networks, whereas the second one works on unipartite graphs. For the first case, we describe the computation of t-invariants (EM), which can be further decomposed by several approaches into disjunctive or overlapping subnetworks. 

We introduce Petri nets as a widely used and suitable formalism to model systems with concurrent processes. In the context of PN, we define the system’s invariants, which give us insight into the dynamic behavior of the system without any kinetic knowledge. To illustrate the idea, we provide a detailed example for the computation of t-invariants (EM) using the Fourier-Motzkin method. From the geometric point of view, t-invariants are equivalent to the extreme rays of a convex cone. We consider the CTI question, which is important to verify a biochemical model. Using the proof by Lipton, we show that this question corresponds to the reachability problem for vector addition systems and is EXPSPACE-hard. 

To consider connectivity-based methods, we define communities. We introduce the Q-modularity measure to verify the partitioning by these algorithms. In addition, we illustrate the methods described, using a small metabolic network, and discuss the development of new methods for the structural analysis of metabolic systems. 

Network reduction plays an important role, in particular, in handling genome-scale networks. We explain how common transition pairs (CTP) and invariant transition pairs (ITP) enable us to compute t-invariants of large networks, even if we will not get a minimal set of t-invariants. Finally, we shortly discuss network verification with respect to kinetic analysis techniques. 

## References

[1] Ravasz E., Somera A.L., Mongru D.A., Oltvai Z.N., Barabási A.L. (2002). Hierarchical organization of modularity in metabolic networks. Science.

[2] Jeong H., Tombor B., Albert R., Oltvai Z.N., Barabási A.L. (2000). The large-scale organization of metabolic networks. Nature.

[3] Hao D., Ren C., Li C. (2012). Revisiting the variation of clustering coefficient of biological networks suggests new modular structure. BMC Syst. Biol..

[4] Reichardt J., Bornholdt S. (2004). Detecting fuzzy community structures in complex networks with a potts model. Phys. Rev. Lett..

[5] Scott J. (1988). Social network analysis. Sociology.

[6] Baldwin C.Y. (2008). Where do transactions come from? Modularity, transactions, and the boundaries of firms. Ind. Corp. Chang..

[7] Adomavicius G., Tuzhilin A. (2005). Toward the next generation of recommender systems: A survey of the state-of-the-art and possible extensions. IEEE Trans. Knowl. Data Eng..

[8] Su X., Khoshgoftaar T.M. (2009). A survey of collaborative filtering techniques. Adv. Artif. Intell..

[9] Zhou M., Venkatesh K. (1999). Modeling, Simulation, and Control of Flexible Manufacturing Systems: A Petri Net Approach. Intelligent Control and Intelligent Automation.

[10] Billington J., Diaz M., Rozenberg G. (1999). Application of Petri Nets to Communication Networks; Lecture Notes in Computer Science.

[11] Kernighan B., Lin S. (1970). An efficient heuristic procedure for partitioning graphs. Bell Syst. Tech. J..

[12] Newman M.E.J., Girvan M. (2004). Finding and evaluating community structure in networks. Phys. Rev. E.

[13] Fortunato S. (2010). Community detection in graphs. Phys. Rep..

[14] Heide H., Bleier L., Steger M., Ackermann J., Dröse J., Schwamb B., Zörnig M., Reichert A., Koch I., Wittig I., Brandt U. (2012). Complexome profiling identifies TMEM126B as a component of the mitochondrial complex I assembly (MCIA) complex. Cell Metab..

[15] Guillaume J.L., Latapy M. (2004). Bipartite structure of all complex networks. Inf. Process. Lett..

[16] Koch I., Reisig W., Schreiber F. (2011). Modeling in Systems Biology: The Petri Net Approach.

[17] Zaitsev D. (2004). Decomposition of Petri nets. Cybern. Syst. Anal..

[18] Zeng Q. (2011). A polynomial-time decomposition algorithm for petri nets based on indexes of transitions. Inf. Technol. J..

[19] Berthelot G., Rozenberg G. (1986). Checking Properties of Nets using Transformations. Advances in Petri Nets 1985.

[20] Berthelot G., Brauer W., Reisig W., Rozenberg G. (1987). Transformations and Decompositions of Nets. Petri Nets: Central Models and Their Properties.

[21] Murata T. Petri Nets: Properties, Analysis and Applications. Proceedings of the IEEE.

[22] Starke P. (1990). Analyse von Petri-Netz-Modellen.

[23] Knuth D. (1997). Fundamental Algorithms.

[24] Cormen T., Leiserson C., Rivest R., Stein C. (2001). Introduction to Algorithms.

[25] Garey M., Johnson D. (1979). Computers and Intractability: A Guide to the Theory of NP-Completness.

[26] Klee V., Minty G. (1972). How good is the simplex algorithm?. Inequalities.

[27] Zadeh N. (1973). A bad network problem for the simplex method and other minimum cost flow algorithms. Math. Progr..

[28] Adleman L. (1994). Molecular computation of solutions to combinatorial problems. Science.

[29] Lipton R. (1995). DNA solution of hard computational problems. Science.

[30] Shor P.W. (1999). Polynomial-time algorithms for prime factorization and discrete logarithms on a quantum computer. Soc. Ind. Appl. Math. Rev..

[31] Paun G., Lingas A., Nilsson B. (2003). Membrane Computing. Fundamentals of Computation Theory.

[32] Kanehisa M., Goto S. (2000). KEGG: Kyoto encyclopedia of genes and genomes. Nucleic Acids Res..

[33] Petri C. (1962). Communication with automata. Ph.D. Thesis 63.

[34] Bryant R. (1995). Binary Decision Diagrams and Beyond: Enabling Technologies for Formal Verification. Proceedings International Conference on Computer Aided Design.

[35] Parikh R. (1966). On context-free languages. J. Assoc. Comput. Mach..

[36] Fourier J. (1826). Solution d’une question particuliére du calcul des inègalitès. In Oeuvres.

[37] Colom J., Silva M. (1991). Convex geometry and semiflows in P/T nets. A comparative study of algorithms for computation of minimal p-semiflows. Lect. Notes Comput. Sci..

[38] Esparza J. (1998). Decidability and complexity of Petri net problems—An introduction. Lect. Notes Comput. Sci..

[39] Schuster S., Hilgetag C. (1994). On elementary flux modes in biochemical reaction systems at steady state. J. Biol. Syst..

[40] Wagner C. (2004). Nullspace approach to determine the elementary modes of chemical reaction systems. J. Phys. Chem. B.

[41] Terzer M., Stelling J. (2008). Large-scale computation of elementary flux modes with bit pattern trees. Bioinformatics.

[42] Jevremovic D., Boley D., Sosa C. Divide-and-Conquer Approach to the Parallel Computation of Elementary Flux Modes in Metabolic Networks. Proceedings of the IEEE International Symposium on Parallel & Distributed Processing.

[43] Lipton R. (1976). The reachability problem requires exponential space.

[44] Schuster S., Pfeiffer T., Moldenhauer F., Koch I., Dandekar T. (2002). Exploring the pathway structure of metabolism: Decomposition into subnetworks and application to *Mycoplasma pneumoniae*. Bioinformatics.

[45] Farkas J. (1902). Theorie der einfachen Ungleichungen. J. für Die Reine Angew. Math..

[46] Ackermann J., Einloft J., Nöthen J., Koch I. (2012). Reduction techniques for network validation in systems biology. J. Theor. Biol..

[47] Koch I., Junker B., Monika Heiner M. (2005). Application of Petri net theory for modelling and validation of the sucrose breakdown pathway in the potato tuber. Bioinformatics.

[48] Liao J.C., Hou S.Y., Chao Y.P. (1996). Pathway analysis, engineering and physiological considerations for redirecting central metabolism. Biotechnol. Bioeng..

[49] Schuster S., Dandekar T., Fell D.A. (1999). Detection of elementary flux modes in biochemical networks: A promising tool for pathway analysis and metabolic engineering. Trends Biotechnolol..

[50] Fischer E., Sauer U. (2003). A novel metabolic cycle catalyzes glucose oxidation and anaplerosis in hungry *Escherichia coli*. J. Biol. Chem..

[51] Klamt S., Gilles E.D. (2004). Minimal cut sets in biochemical reaction networks. Bioinformatics.

[52] Ballerstein K., von Kamp A., Klamt S., Haus U.U. (2012). Minimal cut sets in a metabolic network are elementary modes in a dual network. Bioinformatics.

[53] Sackmann A., Formanowicz D., Formanowicz P., Koch I., Błażewicz J. (2007). An analysis of the Petri net based model of the human body iron homeostasis process. Comput. Biol. Chem..

[54] Grafahrend-Belau E., Schreiber F., Heiner M., Sackmann A., Junker B., Grunwald S., Speer A., Winder K., Ina Koch I. (2008). Modularisation of biochemical networks through hierarchical cluster analysis of T-invariants of biochemical Petri nets. BMC Bioinforma..

[55] Bortfeldt R., Schuster S., Koch I. (2010). Exhaustive analysis of the modular structure of the spliceosomal assembly network: A Petri net approach. In Silico Biol..

[56] Sackmann A. (2005). Modelling and Simulation of signaltransduction pathways of *Saccharomyces crerevisiae* based on Petri net theory. Diploma Thesis.

[57] Sackmann A., Heiner M., Koch I. (2006). Application of Petri net based analysis techniques to signal transduction pathways. BMC Bioinforma..

[58] Grafahrend-Belau E. (2006). Classification of T-invariants in biochemical Petri nets based on different cluster analysis techniques. Master’s Thesis.

[59] Backhaus K., Erichson B., Plinke W., Weiber R. (2003). Multivariate Analysis Methods. An Application-oriented Introduction.

[60] Steinhausen D., Langer K. (1977). Cluster Analysis. An Introduction to Methods for Automatic Classification.

[61] Durbin R., Eddy S., Krogh A., Mitchison G. (1998). Biological Sequence Analysis-Probabilistic Models of Proteins and Nucleic Acids.

[62] Handl J., Knowles J., Kell D.B. (2005). Computational cluster validation in post-genomic data analysis. Bioinformatics.

[63] Rousseeuw P.J. (1987). Silhouettes: A graphical aid to the interpretation and validation of cluster analysis. J. Comput. Appl. Math..

[64] Grunwald S., Speer A., Ackermann J., Koch I. (2008). Petri net modelling of gene regulation of the Duchenne muscular dystrophy. BioSystems.

[65] Pérès S., Beurton-Aimar M., Mazat J.P. (2006). Pathway classification of TCA cycle. IEE Proc. Syst. Biol..

[66] Kaleta C., de Figueiredo L., Schuster S. (2009). Can the whole be less than the sum of its parts? Pathway analysis in genome-scale metabolic networks using elementary flux patterns. Genome Res..

[67] Pfeiffer T., Sánchez-Valdenebro I., Nuño J., Montero F., Schuster S. (1999). METATOOL: For studying metabolic networks. Bioinformatics.

[68] Kholodenko B., Schuster S., Rohwer J., Cascante M., Westerhoff H. (1995). Composite control of cell function:Metabolic pathways behaving as single control units. FEBS Lett..

[69] Rohwer J., Schuster S., Westerhoff H. (1996). How to recognize monofunctional units in a metabolic system. J. Theor. Biol..

[70] Schlegel J. (2012). Network validation and application of Q-modularity to bipartite, directed graphs, in particular Petri nets. Beachelor’s Thesis.

[71] Li C., Donizelli M., Rodriguez N., Dharuri H., Endler L., Chelliah V., Li L., He E., Henry A., Stefan M.I., Snoep J.L., Hucka M., Le Novère N., Laibe C. (2010). BioModels database: An enhanced, curated and annotated resource for published quantitative kinetic models. BMC Syst. Biol..

[72] Gagneur J., Klamt S. (2004). Computation of elementary modes: A unifying framework and the new binary approach. BMC Bioinforma..

[73] Einloft J., Ackermann J., Nöthen J., Koch I. (2013). MonaLisa-visualization and analysis of functional modules in biochemical networks. Bioinformatics.

[74] Murray J. (2002). Mathematical Biology.

[75] Deuflhard P., Bornemann F. (2002). Scientific Computing with Ordinary Differential Equations.

[76] Haken H. (1983). Synergetics: An introduction.

[77] Gardiner C.W. (1985). Handbook of Stochastic Methods.

